# Gut neurotoxin p-cresol induces differential expression of GLUN2B and GLUN2A subunits of the NMDA receptor in the hippocampus and nucleus accumbens in healthy and audiogenic seizure-prone rats

**DOI:** 10.3934/Neuroscience.2020003

**Published:** 2020-03-10

**Authors:** Gigi Tevzadze, Elene Zhuravliova, Tamar Barbakadze, Lali Shanshiashvili, Davit Dzneladze, Zaqaria Nanobashvili, Tamar Lordkipanidze, David Mikeladze

**Affiliations:** 1 4-D Research Institute, Ilia State University, 3/5 Cholokashvili av, Tbilisi, 0162, Georgia; 2 Institute of Chemical Biology, Ilia State University, 3/5 Cholokashvili av, Tbilisi, 0162, Georgia; 3 I. Beritashvili Center of Experimental Biomedicine 14, Gotua Str., Tbilisi 0160, Georgia

**Keywords:** NMDA receptor, p-cresol, epilepsy, nucleus accumbens, hippocampus

## Abstract

Mislocalization and abnormal expression of N-methyl-D-aspartate glutamate receptor (NMDAR) subunits is observed in several brain disorders and pathological conditions. Recently, we have shown that intraperitoneal injection of the gut neurotoxin p-cresol induces autism-like behavior and accelerates seizure reactions in healthy and epilepsy-prone rats, respectively. In this study, we evaluated the expression of GLUN2B and GLUN2A NMDAR subunits, and assessed the activity of cAMP-response element binding protein (CREB) and Rac1 in the hippocampi and nucleus accumbens of healthy and epilepsy-prone rats following p-cresol administration. We have found that subchronic intraperitoneal injection of p-cresol induced differential expression of GLUN2B and GLUN2A between the two brain regions, and altered the GLUN2B/GLUN2A ratio, in rats in both groups. Moreover, p-cresol impaired CREB phosphorylation in both brain structures and stimulated Rac activity in the hippocampus. These data indicate that p-cresol differently modulates the expression of NMDAR subunits in the nucleus accumbens and hippocampi of healthy and epilepsy-prone rats. We propose that these differences are due to the specificity of interactions between dopaminergic and glutamatergic pathways in these structures.

## 1. Introduction

N-methyl-D-aspartate glutamate receptors (NMDARs) play a central role in learning, memory, and synaptic development, and are implicated in various neurological and psychiatric disorders [1],[2]. The function and biophysical features of NMDARs are mainly dictated by their subunit composition. The major variants of the GLUN2 subunit—GLUN2A and GLUN2B—are expressed in a spatio-temporal manner. NMDAR subunit expression and composition are controlled by various mechanisms, such as transcriptional regulation, differential trafficking of receptors, post-transcriptional modifications (e.g. phosphorylation), and protein-protein interactions in membrane macromolecular complexes [3]–[5]. Subcellular localization might be an additional regulator of their physiological and pathological functions, as some NMDA receptors are targeted to synaptic sites while others are inserted extrasynaptically. It is supposed that synaptic localization is more characterized for GLUN2A-containing NMDARs, whereas GLUN2B-enriched NMDARs are found in extrasynaptic membranes of the adult central nervous system (CNS). Different localization and composition is possibly associated with opposite effect on neural cell survival: downstream intracellular cascades of synaptic NMDARs promote cell survival, whereas excessive calcium influx by extrasynaptic NMDARs induces mitochondrial dysfunction and, consequently, cell death [6]. Among specific downstream effectors of synaptic NMDARs, cyclic-AMP response element binding protein (CREB) draws special attention because of its established role in neuronal survival [7],[8]. The inhibitory effect of extrasynaptic NMDAR activation on neuronal viability is mediated by a variety of signaling pathways, including inactivation of CREB and ERK1/2, and expression of pro-apoptotic genes. High surface mobility of GLUN2B-enriched NMDARs (250-fold higher than that of GLUN2A-enriched NMDARs) facilitates their regulated lateral diffusion, endocytosis, and recycling. This mobility process is controlled by several post-translational modifications, including phosphorylation, nitrosylation, and palmitoylation [9]. Mislocalization and abnormal expression and targeting of NMDAR subunits are implicated in several brain disorders and pathological conditions, such as Alzheimer's disease, Parkinson's disease, Huntington's disease, schizophrenia, and stroke (reviewed in [3]).

Membrane delivery and stability of NMDARs depend on the integrity of the actin cytoskeleton [10]. The Rho family of guanosine triphosphatase (GTPase) proteins regulate neuronal structure and function, including neuronal actin dynamics. For example, Rac1, a member of the Rho GTPases, stimulates spine formation, dendrite arborization, elongation, and branching complexity [11]. The expression of Rac1 is significantly increased in the temporal lobe of patients with epilepsy and epilepsy model animals, suggesting that elevated Rac1 expression contributes to the pathophysiology of the disease [12]. In contrast, inhibition of Rac1 activity impairs NMDAR function and induces autism spectrum disorders (ASD)-like social deficits in animals. GLUN2B-containing NMDARs and signals predominantly through Rac promote LTD in hippocampal slices [13]. Activation of GLUN2B-containing NMDARs predominantly activates the downstream Rac/p38 pathway [14],[15].

Gut microbiota modulate host brain function and cognitive behavior, and contribute to the development of neurological disorders [16],[17]. Several species of gut *Clostridium* have been shown to produce a wide range of neurotoxins, including p-cresol—the end product of microbial degradation of tyrosine [18],[19]. P-cresol interferes with the conversion of dopamine to norepinephrine via covalent inactivation of dopamine beta-hydroxylase [20],[21]. Elevated dopamine and reduced norepinephrine levels are consistent with monoamine models of psychopathology, and accumulating evidence supports the role of dopaminergic dysfunction in certain neurological disorders [22].

Nevertheless, the functional link between p-cresol and NMDAR-dependent neurological disorders remains unexplored. Recently, we have shown that intraperitoneal injection of p-cresol induced autism-like behavior in healthy rats and accelerated seizure reactions in epilepsy-prone rats [23],[24]. These alterations were accompanied by the increased expression of GLUN2B in the nucleus accumbens (NAc) [25]. Based on these observations, we hypothesized that p-cresol could promote abnormal subcellular localization of NMDAR subunits and, consequently, alter the composition of synaptic and extrasynaptic NMDARs. In this study, we determined the amount of GLUN2B and GLUN2A, and assessed the activitity of CREB and Rac1 (downstream effectors of NMDARs) in healthy and epilepsy-prone rats following p-cresol treatment. We found that subchronic intraperitoneal injection of p-cresol induced differential expression of the two subunits between the hippocampi and NAc of healthy and epilepsy-prone rats, and altered their GLUN2B/GLUN2A ratio. Furthermore, p-cresol decreased the levels of phosphorylated CREB in both brain structures and stimulated Rac activity in the hippocampus. These data suggest that p-cresol specifically impairs NMDAR-dependent activity in the NAc and hippocampi of healthy and epilepsy-prone rats, and that this effect is mediated via mislocalization of NMDAR subunits.

## 2. Materials and methods

### 2.1. Animals

Healthy Wistar rats and audiogenic seizure-prone Krushinski–Molodkina (KM) rats [26] (160–180 g) were randomly allocated into experimental and control groups, with 5 rats in each group. During the experiments, the rats were allowed water and standard laboratory chow ad libitum, and were maintained under controlled temperature (21–22 °C) and humidity (47 ± 2%), with 12-h light/dark cycle. The rats were housed in cages (transparent polycarbonate, 595 × 380 × 200 mm^3^), with 5 animals per cage. The experimental procedures and animal care and handling were performed in conformity with the European Communities Council Directive EU Directive 2010/63/EU for animal experiments. All experiments were approved by the Institutional Research Projects' Ethics Commission of Ilia State University.

### 2.2. p-cresol administration and isolation of subcellular fractions from brain regions

Rats in the experimental and control groups received daily intraperitoneal injections of p-cresol (30 mg/kg per injection; Sigma-Aldrich) or isotonic saline, respectively, for 21 days. Three days after termination of injections, the animals were sacrificed and decapitated. Immediately after decapitation, their hippocampi and NAc were extracted, rapidly homogenized in an ice-cold buffer (20 mM Tris-HCl (pH 7.4), 0.32 M sucrose, 1 mM EDTA (ethylenediamine tetraacetic acid), 0.5 mM EGTA (ethylene glycol-bis(β-aminoethyl ether)-N,N,N′,N′-tetraacetic acid)) and a cocktail of protease inhibitors (Sigma-Aldrich), and centrifuged at 1000 × g for 10 min. The pellet (nuclear fraction) was collected and the supernatant centrifuged at 12 000 ×g for 15 min. The resulting supernatant (“cytosol fraction”) was collected and stored at −80 °C until further use, while the pellet (membrane fraction) was washed once and centrifuged as before. A concentrated solution of sodium dodecyl sulfate (SDS) was added to the membrane fraction to give a final concentration of 5%, and the sample was incubated at 4 °C for ≥1 h. Protein content of the membrane fraction was determined in quadruplicate using the Micro BCA Protein Assay Kit (cat. no. 23235, Pierce), according the manufacturer's protocol. All samples were stored at −80 °C until analysis.

### 2.3. Western blotting

Aliquots of the solubilized membrane fraction containing 30 µg of total protein in equal volume were applied to SDS gels (4–12%) and electrophoresed. The separated proteins were electroblotted onto 0.45-µm nitrocellulose membranes before the membranes were stained with Ponceau S solution to confirm uniform sample loading and efficient protein transfer. Subsequently, the membranes were blocked with 5% bovine serum albumin (BSA) in Tris-buffered saline with 0.1% Tween-20 (TBST), and incubated for 1h with antibodies against GLUN2B and GLUN2A (sc-390094 and sc-3655R97, 1:1000; Santa Cruz, USA). Following incubation, the membranes were washed in TBST and probed with species-appropriate peroxidase-conjugated affinity-purified secondary antibodies at room temperature for 1 h. After further washing in TBST, standard immunochemical procedures were carried out using chemiluminescent substrate (Santa Cruz, USA). The blots were exposed to X-ray films (Amersham) with intensifying screens to ensure linearity of response. The chemiluminescent bands were acquired and their intensities quantified using Image Lite Studio software.

### 2.4. Real-time polymerase chain reaction (qPCR)

Analysis of copy number variation (CNV) was based on qPCR [27]. DNA was extracted from the nuclear fraction using the Tissue DNA Extraction Kit (GeneON), according to the manufacturer's instructions. The DNA content of the preparations was determined using a NanoDrop spectrophotometer (ThermoFisher, USA) before the samples were diluted to ensure equal DNA concentration in each preparation. *GRIN2A* and *GRIN2B* genes (encoding GLUN2A and GLUN2B, respectively) were amplified by qPCR, using primers whose sequences are listed in Table 1.

Target gene expression was normalized to the housekeeping gene beta-actin. All primers used were purchased from Eurofins. Reverse transcription was performed using the Real-time-PCR Master Mix E3 (GeneON) containing EvaGreen, dUTP, and ROX. qPCR was carried out on QuantStudio 5 Real-Time PCR System (ThermoFisher, USA) using the following thermocycler conditions: 2 min at 95 °C; 40 cycles of 95 °C for 15 s, 59 °C for 30 s, and 72 °C for 33 s. The data were analyzed by comparing cycle threshold (Ct) values.

**Table 1.** Primer sequences used for qPCR.

Target gene	Forward	Reverse
*GRIN2A*	TCCGCCTTTCCGATTTGGG	GCGTCCAACTTCCCAGTTTTC
*GRIN2B*	CTGAGACTGAAGAACAGGAAGATGACCATC	CGGGACTGTATTCCGCATGCAGG

### 2.5. Rac activation assay

Rac activation assay was performed on the “cytosol fraction” containing equal total protein amount, using the Rac1,2,3 G-LISA Activation Assay Kit (Cytoskeleton, cat. no. BK125), according to the manufacturer's protocol. The results were expressed as optical density (OD) per mg of total protein.

### 2.6. Phospho-CREB (pCREB) assay

The levels of Ser133-phosphorylated CREB were assessed in the nuclear fraction using the Human/Mouse/Rat Phospho-CREB (S133) DuoSet IC ELISA Kit (R&D Systems, cat. no. DYC2510-2), according to the manufacturer's protocol. The concentration of pCREB in each sample was calculated from the standard curve run with each assay, and expressed as ng of pCREB per mg of total protein.

### 2.7. Statistical analyses

OD values for GLUN2B and GLUN2A were analyzed with one-way ANOVA. Planned comparisons using two-tailed T-tests were performed to compare the groups. Statistix 9 (Analytical Software, Tallahassee, USA) was used for all statistical analyses.

## 3. Results

**Figure 1.**
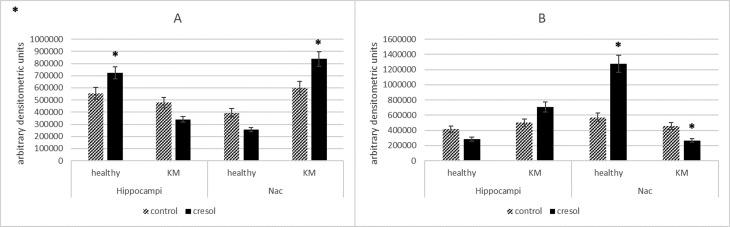
Changes in protein expression of GLUN2A (A) and GLUN2B (B) in the hippocampi and NAc of healthy and KM rats after p-cresol administration, as measured by western blotting. Samples of solubilized membrane fraction with equal amounts of total protein were separated on 4–12% gradient gels. After transfer to 0.45-µm nitrocellulose, the blotted bands were immunodetected with specific primary antibodies, then visualized with peroxidase-labeled secondary IgG antibodies. The bands were acquired and their intensities quantified using Image Lite Studio software. Data are expressed as mean ± SEM and calculated relative to the control group (arbitrarily set at 1). Asterisks indicate statistically significant differences (*P < 0.05).

Brain regions (the hippocampus and NAc) extracted from control and p-cresol-treated rats were used for the following analyses. Abnormal expression of GLUN2B and GLUN2A is implicated in both epilepsy and autism spectrum disorder [28]. Hence, primarily we determined the expression of those two proteins in rat brain tissue after treatment with p-cresol and GLUN2B/GLUN2A ratio were estimated. The results showed that the two subunits were differentially expressed in plasma membranes between the hippocampus and NAc in both healthy and KM rats (Figure 1).

**Figure 2.**
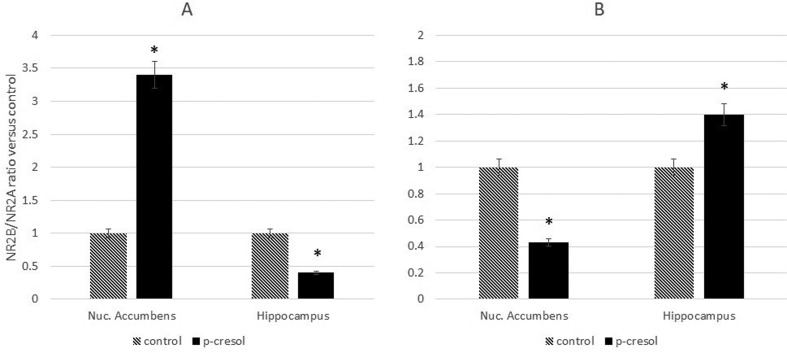
Changes to the GLUN2B/GLUN2A ratio in the hippocampi and NAc of healthy (A) and KM (B) rats after p-cresol administration were detected based on Western Blotting results. Data are expressed as mean ± SEM and calculated relative to the control group (arbitrarily set at 1). Asterisks indicate statistically significant differences (*P < 0.05).

Consequently, the GLUN2B/GLUN2A ratio was altered in both brain structures. In healthy, p-cresol-treated rats, the ratio was decreased in the hippocampus and increased in the NAc (Figure 2A) while the opposite effect was observed in p-cresol-treated KM rats (Figure 2B).

Several genetic studies have been conducted to elucidate the role of *GRIN2A/GRIN2B* genes in the molecular mechanism of the neurological pathologies [29]–[31]. Different copy number variants (CNVs are associated with disorders such as intellectual disability, autism, epilepsy, schizophrenia, and bipolar disorder [32]–[34]. Based on these observations, we performed qPCR to detect CNV gains and losses (deletion/duplication) in *GRIN2A* and *GRIN2B*. No significant differences in Ct (cycle threshold) values between the hippocampus and NAc and between untreated and p-cresol-treated rats were found (data not shown). These results indicate that the number of copies of *GRIN2B* and *GRIN2A* genes is the same in the NAc and hippocampi of healthy and KM rats, and that it is not affected by p-cresol. Thus, it could be suggested that epigenetic regulation of the expression of *GRIN2A/GRIN2B* genes, or post-transcriptional processes, may contribute to the differential spatio-temporal distribution of GLUN2B and GLUN2A NMDAR subunits [29].

**Figure 3.**
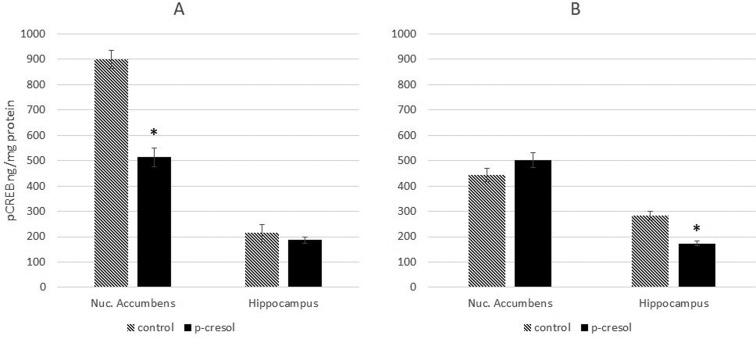
Changes in phosphorylation of CREB in the nuclear fraction of NAc and hippocampi of healthy (A) and KM (B) rats after p-cresol administration. The result shows pCREB amount. Data are expressed in ng pCREB and calculated for mg total protein in each group ±SEM. Asterisks denote statistically significant differences (*P < 0.05).

The high lateral mobility of GLUN2B-containing receptors allows them to translocate between synaptic and extrasynaptic sites, and the GLUN2A/GLUN2B ratios may differ considerably between inputs [6]. Extrasynaptic GLUN1/GLUN2B receptors trigger major cell death pathways, leading to neuronal death [35], while activation of synaptic GLUN2A/GLUN2B-containing receptors may convey pro-survival cues [36] through CREB signaling [6]. Therefore, alterations in the GLUN2B/GLUN2A ratio may be caused by the increased expression of extrasynaptic GLUN2B. In the next experiment, we assessed the activity of CREB. We found that subchronic exposure to p-cresol decreased pCREB levels in the NAc of healthy rats and hippocampi of KM rats, without affecting its levels in the hippocampi of healthy rats and NAc of KM rats (Figure 3). CREB activity was negatively correlated with the amount of GLUN2B protein, suggesting that the observed changes in the GLUN2B/GLUN2A ratio are caused by an increase in extrasynaptic GLUN2B.

**Figure 4.**
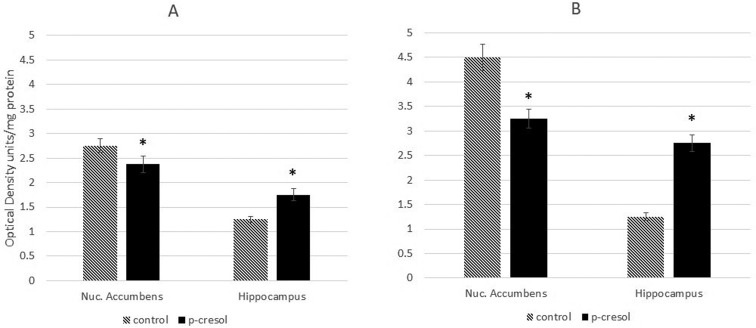
Changes in Rac1 activity in the cytosol fraction of NAc and hippocampi of healthy (A) and KM rats (B) after p-cresol administration. Data are expressed in O.D. as mean ± SEM for each proup and calculated for mg total protein. Asterisks indicate statistically significant differences (*P < 0.05).

The Rho GTPases regulate the actin cytoskeleton and play an important role in the maintenance and reorganization of dendritic spines [37]. Therefore, we next sought to assess the activity of Rac1 in the NAc and hippocampi of healthy and KM rats. The results revealed that, following p-cresol treatment, the levels of active Rac1 were diminished in the NAc and elevated in the hippocampi of rats in both groups (Figure 4).

## 4. Discussion

The hippocampus—the largest structure of the mesial temporal lobe, is usually the primary region of lesions and is responsible for majority of symptoms characterizing temporal lobe epilepsy (TLE). Experimental and clinical data also suggest involvement of the nucleus accumbens (NAс) in propagation of frontal and temporal lobe seizures as well as in behavioral modification [38]. Preclinical studies demonstrated that NAc had been involved in the progress of epilepsy and recurrent epilepsy caused neuronal degeneration via this region-related pathways [39]. It was shown that stimulation of the ventral tegmental area can either inhibit or facilitate the activity of NAc neurons driven by hippocampal input [40]–[42]. Hippocampal glutamatergic pathway in NAc neurons could modify the activity of this region through changes in postsynaptic density, presumably by increasing the number of GLUN2B-containing extrasynaptic NMDARs [43]. Conversely, NMDAR-dependent plasticity in the NAc is required for early stages of learning [44].

We have found that treatment of rats with p-cresol modified the expression of GLUN2B and GLUN2A subunits of NMDAR in the plasma membranes of NAc and hippocampal neurons, significantly changing their GLUN2B/GLUN2A ratio. Importantly, the ratio was altered in opposite directions in the two brain regions: it was increased in the NAc and decreased in the hippocampi of healthy rats, whereas in KM rats, the opposite result was observed. Considering that these changes might have been the result of distinct genomic programmes of synthesis and expression of GLUN2A and GLUN2B subunits in different brain structures, we analysed the copy numbers of *GRIN2A* and *GRIN2B*. No significant differences in the CNV of either gene were found between the NAc and hippocampus, and between untreated and p-cresol-treated rats. Thus, we propose that epigenetic regulation of *GRIN2A/GRIN2B* expression, or post-transcriptional processes, contribute to the differential spatio-temporal distribution of these NMDAR subunits.

To assess the physiological significance of changes in NMDAR subunit composition, we evaluated the activity of CREB—one of the most important signaling-dependent transcription factors whose role in neuronal survival, as well as in synaptic plasticity, neurogenesis, learning, and memory is well-documented [45],[46]. It is thought that GLUN2B localized in the extrasynaptic sites promotes CREB dephosphorylation. In contrast, synaptic NMDARs stimulate CREB phosphorylation [6], which, in turn, initiates synaptogenesis in the hippocampus and NAc [47],[48]. To determine the effect of changed ratio of subunits on the activity of one the key transcription factors–CREB, we have analyzed the phosphorylated form of this protein. We have found that administration of p-cresol impaired CREB phosphorylation in the NAc of healthy rats and hippocampi of KM rats while the level of phosphorylated CREB did not change in the hippocampi of saline-treated rats. An inverse correlation between the activity of CREB and the amount of GLUN2B protein was found, suggesting that elevated GLUN2B/GLUN2A ratio in the NAc of healthy rats and hippocampi of KM rats is due to an increase in extrasynaptic GLUN2B. These data suggest that p-cresol modulates the composition of NMDARs, leading to a reduction in synaptic plasticity in the NAc of normal rats and hippocampi of KM rats.

CREB-responsive genes promote dendritic arborization through stimulation of the Rac pathway [49]; thus, Rac activity should be positively correlated with CREB phosphorylation. However, our results have shown that the two were only correlated in the NAc, and that p-cresol inhibited both Rac activity and CREB phosphorylation. Conversely, in the hippocampus, Rac activity was stimulated by p-cresol, indicating that p-cresol exerts opposing effects on NAc and hippocampal cells.

The effects of p-cresol on the hippocampi and NAc of healthy and epilepsy-prone rats might be caused by the different anatomical and biochemical features of these structures. Considering that p-cresol alters dopamine metabolism, we propose that the variable effects of this toxin on the hippocampus and NAc are mediated by differences in the density and activity of dopaminergic pathways in the two brain regions. Both D_1_ and D_2_ dopamine receptors physically interact with NMDAR subunits, and dopaminergic ligands dynamically regulate this complex. Surface expression of NR1 and GLUN2B proteins was reported to increase in cultured prefrontal cortex neurons following exposure to a D1DR (D_1_ dopamine receptors) agonist [50],[51]. Additionally, D_1_ receptors mediate CREB phosphorylation via phosphorylation of NMDARs [52]. In a membrane fractionation study involving striatal tissue homogenates, activation of the D_1_ receptor triggered redistribution of NR1, GLUN2A, and GLUN2B to synaptosomal compartments [53]. Thus, we suppose that surface expression, and possibly redistribution, of NMDAR subunits in p-cresol-exposed brain depends on its dopamine concentration and on the docking of NMDAR subunits by dopamine receptor subtypes.

The changes to glutamate receptor composition in the hippocampi of epilepsy-prone, audio-sensitive rats appear to be the result of genetically determined variations in dopaminergic and glutamatergic systems in the brain. The striatal dopamine system of KM rats is different to that of healthy rats. Basal dopamine levels are 25% higher in brain dialysates of KM rats than in those of control rats, while D_2_ and NMDAR densities are compensatorily lowered [26]. Thus, the higher dopamine concentration in KM rats could contribute to the different effect of p-cresol on KM and healthy rats.

Chernigovskaya and colleagues [54] revealed that expression of VGLUT2 (glutamate transporter) and GLUN2B in KM rats increased until day 14 after birth, before dramatically decreasing in later stages of life. The genetically determined increase inERK1/2 signaling was also observed during postnatal ontogenesis of KM rats. The latter effect could be related to disturbances in the synthesis and activity of proteins regulating hippocampal glutamatergic transmission during the development of seizure susceptibility.

Collectively, our data suggest that interactions between the glutamatergic and dopaminergic systems play a central role in synaptic plasticity in the NAc and hippocampus exposed to the gut neurotoxin p-cresol. Elevation of dopamine levels in response to inhibition of dopamine beta-hydroxylase can alter the surface expression and subcellular localization of the DR-NMDA heteroreceptor in these brain regions, leading to changes in downstream effector activity and abnormal extrapyramidal motor and cognitive behaviors.

## 5. Conclusions

Subchronic intraperitoneal injection of p-cresol caused differential expression of GLUN2B and GLUN2A between murine hippocampi and NAc, and altered the GLUN2B/GLUN2A ratio differently in healthy and KM rats. These changes were accompanied by decreased CREB phosphorylation in both brain structures, and increased Rac activitiy in the hippocampus. These data suggest that p-cresol has distinct effects on NMDAR-dependent activity in the NAc and hippocampus in healthy and epilepsy-prone rats.

## References

[1] Yamamoto H, Hagino Y, Kasai S (2015). Specific roles of NMDA receptor subunits in mental disorders. Curr Mol Med.

[2] Zhou Q, Sheng M (2013). NMDA receptors in nervous system diseases. Neuropharmacology.

[3] Sanz-Clemente A, Nicoll RA, Roche KW (2013). Diversity in NMDA receptor composition: many regulators, many consequences. Neuroscientist.

[4] Mao LM, Guo ML, Jin DZ (2011). Post-translational modification biology of glutamate receptors and drug addiction. Front Neuroanat.

[5] Koster KP, Francesconi W, Berton F (2019). Developmental NMDA receptor dysregulation in the infantile neuronal ceroid lipofuscinosis mouse model. Elife.

[6] Hardingham GE, Bading H (2010). Synaptic versus extrasynaptic NMDA receptor signalling: Implications for neurodegenerative disorders. Nat Rev Neurosci.

[7] Monti B, Marri L, Contestabile A (2002). NMDA receptor-dependent CREB activation in survival of cerebellar granule cells during in vivo and in vitro development. Eur J Neurosci.

[8] Lee B, Butcher GQ, Hoyt KR (2005). Activity-dependent neuroprotection and cAMP response element-binding protein (CREB): Kinase coupling, stimulus intensity, and temporal regulation of CREB phosphorylation at serine 133. J Neurosci.

[9] Chen BS, Roche KW (2009). Growth factor-dependent trafficking of cerebellar NMDA receptors via protein kinase B/Akt phosphorylation of NR2C. Neuron.

[10] Duffney LJ, Wei J, Cheng J (2013). Shank3 deficiency induces NMDA receptor hypofunction via an actin-dependent mechanism. J Neurosci.

[11] Ridley AJ (2006). Rho GTPases and actin dynamics in membrane protrusions and vesicle trafficking. Trends Cell Biol.

[12] Li J, Xing H, Jiang G (2016). Increased expression of Rac1 in epilepsy patients and animal models. Neurochem Res.

[13] Liu L, Wong TP, Pozza MF (2004). Role of NMDA receptor subtypes in governing the direction of hippocampal synaptic plasticity. Science.

[14] Krapivinsky G, Krapivinsky L, Manasian Y (2003). The NMDA receptor is coupled to the ERK pathway by a direct interaction between NR2B and RasGRF1. Neuron.

[15] Liu XY, Chu XP, Mao LM (2006). Modulation of D2R-NR2B interactions in response to cocaine. Neuron.

[16] Hsiao EY, McBride SW, Hsien S (2013). Microbiota modulate behavioral and physiological abnormalities associated with neurodevelopmental disorders. Cell.

[17] Thakur AK, Shakya A, Husain GM (2014). Gut-Microbiota and mental health: current and future perspectives. J Pharmacol Clin Toxicol.

[18] Persico AM, Napolioni V (2013). Autism genetics. Behav Brain Res.

[19] Nicholson JK, Holmes E, Kinross J (2012). Host-gut microbiota metabolic interactions. Science.

[20] Goodhart PJ, DeWolf WE, Kruse LI (1987). Mechanism-based inactivation of dopamine .beta.-hydroxylase by p-cresol and related alkylphenols. Biochemistry.

[21] Southan C, DeWolf WE, Kruse LI (1990). Inactivation of dopamine β-hydroxylase by p-cresol: Evidence for a second, minor site of covalent modification at tyrosine 357. Biochim Biophys Acta.

[22] Pavăl D (2017). A dopamine hypothesis of autism spectrum disorder. Dev Neurosci.

[23] Tevzadze G, Oniani N, Zhuravliova E (2018). Effects of a gut microbiome toxin, p-cresol, on the indices of social behavior in rats. Neurophysiology.

[24] Tevzadze G, Nanobashvili Z, Zhuravliova E (2018). Effects of a gut microbiome toxin, p-Cresol, on the susceptibility to seizures in rats. Neurophysiology.

[25] Tevzadze G, Zhuravliova E, Meparishvili M (2019). Effects of a Gut Microbiome Toxin, p-Cresol, on the Contents of the NMDA2B Receptor Subunit in the Nucl. Accumbens of Rats. Neurophysiology.

[26] Poletaeva II, Surina NM, Kostina ZA (2017). The Krushinsky-Molodkina rat strain: The study of audiogenic epilepsy for 65 years. Epilepsy Behav.

[27] Ma L, Chung WK (2014). Quantitative analysis of copy number variants based on real-time lightcycler PCR. Curr Protoc Hum Genet.

[28] Ishii A, Hirose S (2017). New genes for epilepsy–autism comorbidity. J Pediatr Neurol.

[29] Ryley PR, Albertson AJ, Buckingham SC (2013). Status epilepticus triggers early and late alterations in brain-derived neurotrophic factor and NMDA glutamate receptor Grin2b DNA methylation levels in the hippocampus. Neuroscience.

[30] Paoletti P, Bellone C, Zhou Q (2013). NMDA receptor subunit diversity: Impact on receptor properties, synaptic plasticity and disease. Nat Rev Neurosci.

[31] Endele S, Rosenberger G, Geider K (2010). Mutations in GRIN2A and GRIN2B encoding regulatory subunits of NMDA receptors cause variable neurodevelopmental phenotypes. Nat Genet.

[32] Sebat J, Levy DL, McCarthy SE (2009). Rare structural variants in schizophrenia: one disorder, multiple mutations; one mutation, multiple disorders. Trends Genet.

[33] Weiss LA, Shen Y, Korn JM (2008). Association between microdeletion and microduplication at 16p11.2 and autism. N Engl J Med.

[34] Coe BP, Witherspoon K, Rosenfeld JA (2014). Refining analyses of copy number variation identifies specific genes associated with developmental delay. Nat Genet.

[35] Gladding CM, Raymond LA (2011). Mechanisms underlying NMDA receptor synaptic/extrasynaptic distribution and function. Mol Cell Neurosci.

[36] Chen M, Lu TJ, Chen XJ (2008). Differential roles of NMDA receptor subtypes in ischemic neuronal cell death and ischemic tolerance. Stroke.

[37] Nakayama AY, Harms MB, Luo L (2000). Small GTPases Rac and Rho in the maintenance of dendritic spines and branches in hippocampal pyramidal neurons. J Neurosci.

[38] Kowski AB, Voges J, Heinze HJ (2015). Nucleus accumbens stimulation in partial epilepsy - A randomized controlled case series. Epilepsia.

[39] Fu J, Liu Y, Yang K (2018). Effect of accumbens nucleus shell lesioning on bitemporal lobe epilepsy in rat model. Folia Neuropathol.

[40] Yang CR, Mogenson GJ (1984). Electrophysiological responses of neurones in the nucleus accumbens to hippocampal stimulation and the attenuation of the excitatory responses by the mesolimbic dopaminergic system. Brain Res.

[41] Pennartz CMA, Dolleman-Van der Weel MJ, Lopes da Silva FHL (1992). Differential membrane properties and dopamine effects in the shell and core of the rat nucleus accumbens studied in vitro. Neurosci Lett.

[42] Gonon F, Sundstrom L (1996). Excitatory effects of dopamine released by impulse flow in the rat nucleus accumbens in vivo. Neuroscience.

[43] Shen H, Moussawi K, Zhou W (2011). Heroin relapse requires long-term potentiation-like plasticity mediated by NMDA2b-containing receptors. Proc Natl Acad Sci U S A.

[44] Vega-Villar M, Horvitz JC, Nicola SM (2019). NMDA receptor-dependent plasticity in the nucleus accumbens connects reward-predictive cues to approach responses. Nat Commun.

[45] Ortega-Martínez S (2015). A new perspective on the role of the CREB family of transcription factors in memory consolidation via adult hippocampal neurogenesis. Front Mol Neurosci.

[46] Sakamoto K, Karelina K, Obrietan K (2011). CREB: A multifaceted regulator of neuronal plasticity and protection. J Neurochem.

[47] Marie H, Morishita W, Yu X (2005). Generation of silent synapses by acute in vivo expression of CaMKIV and CREB. Neuron.

[48] Brown TE, Lee BR, Mu P (2011). A silent synapse-based mechanism for cocaine-induced locomotor sensitization. J Neurosci.

[49] Wayman GA, Impey S, Marks D (2006). Activity-dependent dendritic arborization mediated by CaM-kinase I activation and enhanced CREB-dependent transcription of Wnt-2. Neuron.

[50] Gao C, Wolf ME (2008). Dopamine receptors regulate NMDA receptor surface expression in prefrontal cortex neurons. J Neurochem.

[51] Hu JL, Liu G, Li YC (2010). Dopamine D1 receptor-mediated NMDA receptor insertion depends on Fyn but not Src kinase pathway in prefrontal cortical neurons. Mol Brain.

[52] Dudman JT, Eaton ME, Rajadhyaksha A (2003). Dopamine D1 receptors mediate CREB phosphorylation via phosphorylation of the NMDA receptor at Ser897-NR1. J Neurochem.

[53] Dunah AW, Sirianni AC, Fienberg AA (2004). Dopamine D1-dependent trafficking of striatal N-Methyl-D-aspartate glutamate receptors requires Fyn protein tyrosine kinase but not DARPP-32. Mol Pharmacol.

[54] Chernigovskaya EV, Korotkov AA, Dorofeeva NA (2019). Delayed audiogenic seizure development in a genetic rat model is associated with overactivation of ERK1/2 and disturbances in glutamatergic signaling. Epilepsy Behav.

